# Oncogenic *transgelin-2* is differentially regulated in *isocitrate dehydrogenase* wild-type vs. mutant gliomas

**DOI:** 10.18632/oncotarget.26365

**Published:** 2018-12-14

**Authors:** Sasha J. Beyer, Erica H. Bell, Joseph P. McElroy, Jessica L. Fleming, Tiantian Cui, Aline Becker, Emily Bassett, Benjamin Johnson, Pooja Gulati, Ilinca Popp, Ori Staszewski, Marco Prinz, Anca L. Grosu, Saikh Jaharul Haque, Arnab Chakravarti

**Affiliations:** ^1^ Department of Radiation Oncology, Arthur G. James Hospital/The Ohio State University Comprehensive Cancer Center, Columbus, OH, USA; ^2^ Center for Biostatistics, The Ohio State University, Columbus, OH, USA; ^3^ Department of Radiation Oncology, Medical Center University of Freiburg, Freiburg, Germany; ^4^ German Cancer Consortium (DKTK), Partner Site, Freiburg, Germany; ^5^ Institute of Neuropathology, Medical Faculty, University of Freiburg, Freiburg, Germany; ^6^ BIOSS Centre for Biological Signaling Studies, University of Freiburg, Freiburg, Germany; ^7^ CIBSS Centre for Integrative Biological Signaling Studies, University of Freiburg, Freiburg, Germany

**Keywords:** transgelin-2, glioma, glioblastoma, isocitrate dehydrogenase (IDH1/2) mutation, invasion

## Abstract

The presence of an *isocitrate dehydrogenase* (*IDH1/2*) mutation in gliomas is associated with favorable outcomes compared to gliomas without the mutation (*IDH1/2* wild-type, WT). The underlying biological mechanisms accounting for improved clinical outcomes in *IDH1/2* mutant gliomas remain poorly understood, but may, in part, be due to the glioma CpG island methylator phenotype (G-CIMP) and epigenetic silencing of genes. We performed profiling of *IDH1/2* WT versus *IDH1/2* mutant Grade II and III gliomas and identified *transgelin-2* (*TAGLN2*), an oncogene and actin-polymerizing protein, to be expressed at significantly higher levels in *IDH1/2* WT gliomas compared to *IDH1/2* mutant gliomas. This differential expression of *TAGLN2* was primarily due to promoter hypermethylation in *IDH1/2* mutant gliomas, suggesting involvement of *TAGLN2* in the G-CIMP. Our results also suggest that *TAGLN2* may be involved in progression due to higher expression in glioblastomas compared to *IDH1/2* WT gliomas of lower grades. Furthermore, our results suggest that *TAGLN2* functions as an oncogene by contributing to proliferation and invasion when overexpressed in *IDH1/2* WT glioma cells. Taken together, this study demonstrates a possible link between increased *TAGLN2* expression, invasion and poor patient outcomes in *IDH1/2* WT gliomas and identifies *TAGLN2* as a potential novel therapeutic target for *IDH1/2* WT gliomas.

## INTRODUCTION

Grade IV glioblastomas (GBM) demonstrate dismal prognoses due to their aggressive nature and resistance to standard of care therapies. Despite multi-modality treatment with maximal safe resection, chemotherapy and radiation, the median overall survival for these patients is only 12-15 months [1, 2]. In 2008, the *IDH1/2* mutation was discovered in gliomas [3] and has been associated with improved prognoses in gliomas independent of tumor grade (Grade II-IV) or histologic subtype (astrocytoma, oligodendroglioma, oligoastrocytoma) [4]. The World Health Organization (WHO) revised the low(er) grade glioma (LGG) classification system in 2016 to include *isocitrate dehydrogenase* (*IDH1/2)* mutations and *1p/19q* co-deletion status in addition to glioma grade and histology [5]. As a result, *IDH1/2* mutation status is routinely being utilized in the clinic to help predict tumor prognosis and guide management decisions for glioma patients [6]. While *IDH1/2* mutations most commonly occur in Grade II and III gliomas, they are also present in approximately 5% of GBM that have progressed from lower grade gliomas, known as secondary GBM [3, 7]. Regardless of the grade, *IDH1/2* mutations are a favorable prognostic factor amongst all gliomas. In fact, secondary GBM patients harboring an *IDH1/2* mutation (median survival of 2.1 years) often have improved survival compared to LGG without the mutation, referred to as *IDH1/2* wild-type (*IDH1/2* WT) (median survival of 1.7 years) [4, 8, 9].

The *IDH1/2* enzyme catalyzes the oxidative decarboxylation of isocitrate to form α-ketoglutarate (α-KG), an important reaction for glutamine metabolism, lipogenesis, and regulation of cellular redox status in the cell [10]. *IDH1* and *IDH2* proteins are encoded by separate genes that share approximately 70% sequence homology in humans [10]. The heterozygous R132H mutation in *IDH1* accounts for 95% of *IDH1/2* mutations in gliomas [11], however heterozygous mutations also occur in the analogous amino acid of *IDH2 (*R172), including R172G, R172K, and R172M [4, 12]. Both the R132 and R172 residues are located at the active site of *IDH1/2* and form hydrogen bonds with the isocitrate substrate, therefore decreasing its binding affinity. As a result, IDH1 and IDH2 mutations compromise α-KG production, but at the same time result in neomorphic enzymatic activity that reduces α-KG to 2-hydroxyglutarate (2-HG). The mechanism by which aberrant 2-HG causes oncogenesis is poorly understood. It has been well-established that 2-HG inhibits dioxygeases, including DNA demethylases, that result in a global hypermethylated phenotype and gene silencing, known as glioma CpG-island hypermethylator phenotype (G-CIMP) [13]. The specific gene expression changes associated with this glioma hypermethylated phenotype in *IDH1/2* mutant gliomas are yet to be elucidated. In this study, we performed molecular profiling of *IDH1/2* WT versus mutant low(er) grade II and III gliomas to identify key molecular pathways that may contribute to differences in survival outcomes. To this end, by employing an mRNA and proteomics profiling approach, we identified *transgelin-2* (*TAGLN2)* to be differentially expressed between *IDH1/2* WT and mutant gliomas.

*TAGLN2* is an actin-polymerizing protein important for regulation of the actin cytoskeleton during normal physiologic processes, including cell proliferation, differentiation, migration, and immunologic synapse formation (reviewed in [14–16]). *TAGLN2* and *transgelin-*3 (*TAGLN3*) are both homologues of *transgelin-1 (TAGLN1)*, a marker of smooth muscle cell differentiation, that appears to be widely expressed in tissues with similar biological functions as its homologues [15]. *TAGLN2* is highly expressed in epithelial cells, while *TAGLN3* is neuron-specific [14].

*TAGLN2* has also been shown to be dysregulated in multiple cancer types, such as colorectal [15, 17, 18], breast [19, 20], cervical [21], lung [22, 23], hepatocellular [24, 25], pancreatic [26, 27], head and neck [28], meningiomas [29] and more recently gliomas [30]. While the role of *TAGLN2* in cancers has been controversial, multiple studies have shown that *TAGLN2* contributes to invasion, cell proliferation, metastases, treatment resistance and poor prognosis among several cancer types, however little is known about its role in glioma biology. Han et al. [30] recently reported that silencing of *TAGLN2* decreases proliferation and invasion in gliomas and that *TAGLN2* may be a prognostic biomarker in gliomas. Thus, it remains to be elucidated how the *IDH1/2* mutant-generated oncometabolite, 2-HG, regulates the expression of *TAGLN2* and how *TAGLN2* in turn drives glioma cell proliferation and invasion.

Here, we have shown that *TAGLN2* functions as an oncogene in *IDH1/2* WT gliomas and is expressed at significantly higher levels in *IDH1/2* WT gliomas due to *TAGLN2* promoter hypermethylation and subsequent gene silencing in *IDH1/2* mutant gliomas. We also found higher mRNA expression of *TAGLN2* in GBM compared to *IDH1/2* WT gliomas of lower grades. An in-depth understanding of the role and regulation of *TAGLN2* in gliomas may be useful in identifying novel therapeutic vulnerabilities in *TAGLN2*-associated signaling pathways driving proliferation, invasion and therapeutic resistance mechanisms in gliomas.

## RESULTS

### *IDH1/2* WT LGGs have increased *TAGLN2* expression compared to *IDH1/2* mutant gliomas and *TAGLN2* mRNA expression correlates with glioma grade

**Figure 1:**
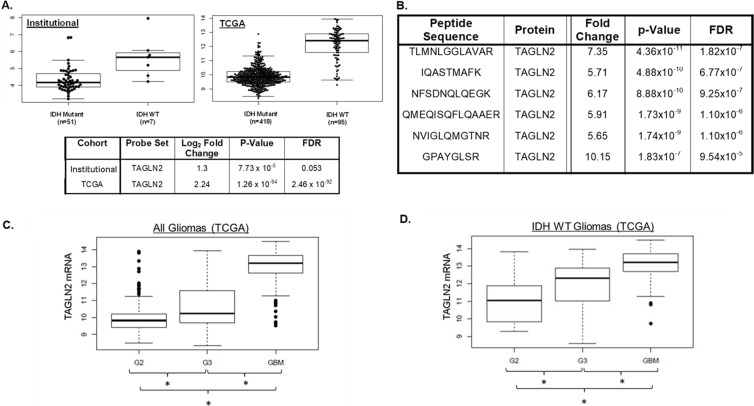
*IDH1/2* WT LGGs have increased *TAGLN2* expression compared to *IDH1/2* mutant gliomas and TAGLN2 mRNA expression correlates with glioma grade **(A)** *TAGLN2* mRNA was expressed at significantly higher levels in *IDH1/2* WT tumors compared to *IDH1/2* mutant tumors in both our institutional (p-value=7.73×10^−5^; FDR=0.053) and the validation TCGA (p-value <0.0001; FDR < 0.0001) cohorts. **(B)** Mass spectrometry identified higher *TAGLN2* protein expression in *IDH1/2* WT compared to *IDH1/2* mutant LGG from our institutional cohort. Six different peptides corresponding to *TAGLN2* protein were expressed at significantly up-regulated in *IDH1/2* WT compared to *IDH1/2* mutant tumors. **(C)** Publicly available *TAGLN2* mRNA expression of all Grade II (G2, n=249), III (G3, n=265) and IV (GBM, n=153) gliomas from the TCGA database are significantly different (p<0.0001). **(D)** *TAGLN2* expression in *IDH1/2* WT Grade II (n=21), III (n=52) and IV (n=133) tumors from TCGA data was significantly different (p=5.136×10^−20^).

In order to better understand the biological mechanisms responsible for differences in clinical outcomes between patients harboring *IDH1/2* WT and *IDH1/2* mutant gliomas, we performed mRNA profiling to compare gene expression between *IDH1/2* WT (n=7) and *IDH1/2* mutant (n=51) grade II and III gliomas from our institutional cohort. Among the 175 significantly differentially expressed genes, we identified *TAGLN2* as an upregulated gene in *IDH1/2* WT grade II and III gliomas compared to *IDH1/2* mutant tumors (p-value=7.73×10^−5^; FDR=0.053) in our institutional cohort (Figure 1A). We confirmed the up-regulation of *TAGLN2* mRNA levels in *IDH1/2* WT tumors (n=95) compared to *IDH1/2* mutant tumors (n=418) using the publicly available TCGA LGG database (p-value < 0.0001; FDR=< 0.0001) (Figure 1A).

In addition, we performed proteomic profiling of an institutional discovery cohort of 40 LGGs (7 *IDH1*/2 WT and 33 *IDH1/2 mutant*) that identified 120 differentially expressed peptides in *IDH1/2* WT vs. *IDH1/2* mutant tumors that mapped to 65 proteins (FDR p value <0.10). Interestingly, 6 of the 120 significantly differentially regulated peptides corresponded to *TAGLN2* protein (Figure 1B). These six *TAGLN2* peptides were significantly upregulated in *IDH1/2* WT compared to *IDH1/2* mutant LGGs with fold-changes ranging from 5.65 to 10.15 (Figure 1B). We validated these findings in an additional institutional cohort of *IDH1/2* WT (n=7) versus *IDH1/2* mutant (n=23) grade II and III gliomas. We identified 36 significantly differentially regulated proteins in common with our discovery cohort, including 3 peptides corresponding to *TAGLN2* (Supplemental Figure 1). Unfortunately, *TAGLN2* was not included in the reverse-phase protein lysate array (RPPA) used in the TCGA analysis and therefore *TAGLN2* protein levels could not be validated in tumors from the TCGA cohort [8].

Because *TAGLN2* mRNA and protein were both overexpressed in *IDH1/2* WT LGGs and ~90% of GBMs carry *IDH1/2* WT, we assessed *TAGLN2* mRNA expression levels in malignant GBMs. We compared *TAGLN2* gene expression levels among all grade II (n=249) and grade III (n=265) gliomas as well as GBMs (n=153) included in the TCGA database. Our analyses showed that not only was *TAGLN2* expressed at significantly higher levels in GBMs compared to LGGs, but *TAGLN2* was found to be significantly associated with glioma grade (p< 0.0001) (Figure 1C). In order to confirm that this correlation was not due to the association between grade and *IDH1/2* status, we also evaluated *TAGLN2* mRNA levels in *IDH1/2* WT gliomas of all grades. *TAGLN2* mRNA levels were significantly different between *IDH1/2* WT grade II (n=21), grade III (n=52) and grade IV (n=133) gliomas from the TCGA database (p< 0.0001) (Figure 1D). It is important to note, however, that *TAGLN2* gene expression data in TCGA GBM and LGG cohorts were acquired at different times, which may also account for differences in *TAGLN2* gene expression.

### Down-regulation of *TAGLN2* expression in *IDH1/2* mutant gliomas correlates with promoter hypermethylation

**Figure 2:**
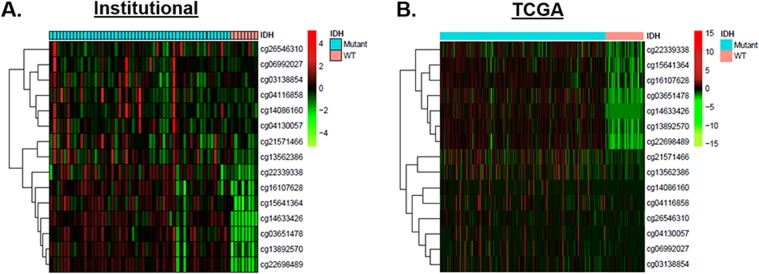
Increased *TAGLN2* promoter hypermethylation accounts for decreased *TAGLN2* expression in *IDH1/2* mutant gliomas **(A)** Promoter methylation was detected in *IDH1/2* mutant tumors (n=54, cyan) and *IDH1/2* WT tumors (n=8, salmon) from our institutional cohort using 15 CpG *TAGLN2* promoter methylation sites included on the Illumina HM-450K array. *IDH1/2* mutant showed significantly higher levels of methylation (FDR<0.05) in the majority of CpG islands corresponding to *TAGLN2* (n=11), as demonstrated by the heat map. Low methylation levels are denoted in green and high methylation levels are denoted in red. **(B)** Methylation results were validated using methylation data from the publicly available TCGA cohort.

While the association between the hypermethylator phenotype and *IDH1/2* mutation in gliomas has been well-established, the majority of individual genes involved remain to be identified. Since *TAGLN2* was down-regulated in *IDH1/2* mutant gliomas, we examined our global methylation data to determine whether increased promoter methylation may account for decreased *TAGLN2* expression in these tumors. Indeed, 60% (n=9/15) of the 15 CpG sites predicted to correspond to the *TAGLN2* promoter showed significantly higher levels of methylation (FDR<0.05) in *IDH1/2* mutant tumors. The heatmap in Figure 2A depicts methylation intensity of each of the 15 CpG sites corresponding to TAGLN2 on the array. These results were validated using methylation data from the publicly available TCGA cohort (Figure 2B).

### Poor prognosis associated with *TAGLN2* expression is dependent on *IDH1/2* mutation status in gliomas

**Figure 3:**
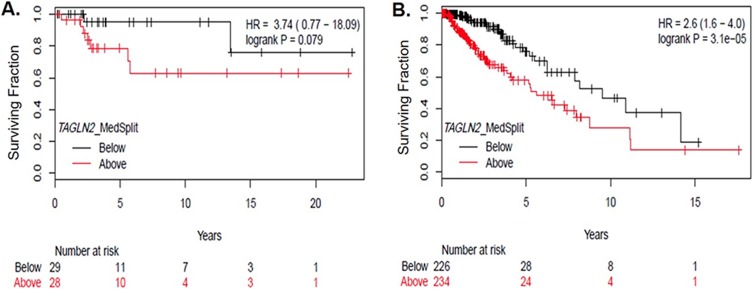
*TAGLN2* mRNA levels are associated with poor survival among gliomas **(A)** *TAGLN2* mRNA levels above the median (detected by Affymetrix Clariom D array) trended toward poor OS in lower grade gliomas from our institutional cohort (HR 3.74, p=0.079), as shown by the Kaplan Meier curve. **(B)** In the TCGA validation dataset, high *TAGLN2* mRNA levels above the median were significantly associated with poor survival (HR= 2.6, p=0.00003).

Since *TAGLN2* has been shown to be a poor prognostic biomarker in other cancers and may play a role in glioma progression, we determined whether *TAGLN2* expression was associated with overall survival (OS) of patients in both institutional and TCGA glioma cohorts. As shown in the Kaplan-Meier curve in Figure 3A, patients with high *TAGLN2* mRNA levels (median split) trended toward worse OS in our institutional cohort of 58 grade II/III patients (HR 3.74, 0.77-18.09, p=0.079). In the LGG-TCGA validation cohort (n=513), patients with *TAGLN2* mRNA above the median had significantly worse OS (HR 2.6, 1.6-4.0, p= 3.1×10^−5^) than patients with mRNA levels below the median (Figure 3B). While the trend was similar between *TAGLN2* expression and OS in both LGG cohorts, our institutional cohort did not reach statistical significance, which may be due to the limited number of survival events in this cohort.

In order to further assess the prognostic significance of *TAGLN2*, we categorized *TAGLN2* mRNA expression as above or below median and correlated expression with clinical/pathologic characteristics from our institutional and TCGA LGG cohorts (Table 1). The TCGA analysis confirmed our previous results that increased *TAGLN2* expression was significantly associated with *IDH1/2* WT and higher grade gliomas (Table 1). Other clinical-pathologic factors known to be associated with *IDH1/2* WT gliomas, such as *1p/19q* non-codeletion status, astrocytoma histology and older age, were also identified to significantly correlate with *TAGLN2* expression in the TCGA analysis (Table 1). While there was a trend toward increased *TAGLN2* mRNA expression in gliomas that are *IDH1/2* WT, *1p/19q* non-codeleted and astrocytomas in our institutional cohort (Table 1), an inadequate sample number could again explain why the association did not reach statistical significance.

**Table 1:** Clinical-pathological characteristics of patients analyzed for *TAGLN2* mRNA expression in institutional and TCGA LGG cohorts

Clinical Factor	Institutional Cohort *TAGLN2* Expression			TCGA Cohort *TAGLN2* Expression		
*TAGLN2* mRNA	Above median (n=29)	Below median (n=29)	P	Above median (n=258)	Below median (n=258)	P
**Age**	42	42	**0.539**	45	38	**<0.001**
**Gender**			**0.038**			**0.182**
Female	4 (13.8%)	12 (41.4)		101 (39/1%)	100 (38.8%)	
Male	25 (86.2%)	17 (58.6%)		134 (51.9%)	122 (47.3%)	
Unknown	N/A	N/A		23 (8.9%)	36 (14.0%)	
**Histology**			**0.278**			**<0.001**
Astrocytoma	7 (24.1%)	4 (13.8%)		106 (41.1%)	63 (24.4%)	
Mixed	13 (44.8%)	13 (44.8%)		56 (21.7%)	58 (22.5%)	
Oligodendroglioma	7 (24.1%)	12 (41.4%)		73 (28.3%)	101 (39.1%)	
Unknown	2 (6.9%)	N/A				
**Grade**			**0.263**			**<0.001**
II	12 (41.4%)	7 (24.1%)		92 (35.7%)	124 (48.1%)	
III	17 (58.6%)	22 (75.9%)		143 (55.4%)	98 (38.0%)	
Unknown	N/A	N/A		23 (8.9%)	36 (14.0%)	
***IDH1/2* Mutation Status**			**0.102**			**<0.001**
IDH1/2 Mutant	23 (79.3%)	28 (96.6%)		168 (65.1%)	251 (97.3%)	
IDH1/2 WT	6 (20.7%)	1 (3.4%)		88 (34.1%)	6 (2.3%)	
Unknown	N/A	N/A		2 (0.8%)	1 (0.4%)	
**1p/19q Status**			**0.114**			**0.005**
Co-deleted	11 (37.9%)	18 (62.1%)		69 (26.7%)	100 (38.8%)	
Non-co-deleted	18 (62.1%)	11 (37.9%)		189 (73.3%)	158 (61.2%)	

A multivariate analysis of the TCGA data was also performed to determine whether *TAGLN2* is a prognostic biomarker independent of other clinical variables. However, after adjusting for age, gender, histology, grade, *IDH1/2* mutation status and *1p/19q* co-deletion status in our multivariate analysis, *TAGLN2* expression was no longer significantly associated with OS (Table 2), which may be due to its strong association with *IDH1/2* mutation status and older age (Table 1). Since *IDH1/2* mutation status has also shown to be highly associated with younger age in adults with LGGs [7], *IDH1/2* mutation status also lost significance in our multivariate analysis. Unfortunately, there were insufficient survival events available from the institutional cohort to perform a multivariate analysis comparing *TAGLN2* expression with OS.

**Table 2:** Multi-variable analysis of clinical-pathological factors with OS from low(er) grade gliomas in the TCGA cohort

Variable	HR	95% CI	P
Gender	1.035	0.58-1.85	0.907
Age at diagnosis (continuous)	1.049	1.022-1.077	<0.001^*^
Histology			
Astrocytoma	0.98	0.48-2.00	0.957
Oligodendroglioma	0.55	0.23-1.28	0.165
*IDH1/2* WT (non-mutated)	2.37	0.75-7.50	0.142
1p/19q Non-codeleted	2.25	0.84-6.05	0.108
*TAGLN2* mRNA	1.25	0.83-1.86	0.285

### *TAGLN2* regulates proliferation of *IDH1/2* WT GBM cells *in vitro*

Since *TAGLN2* was shown to be associated with poor prognosis and tumor progression, we evaluated the role of *TAGLN2* in cell proliferation *in vitro*. Our *in vitro* studies were conducted using high grade glioma GBM30 neurospheres (primary) and U87 MG established cell lines since we found that GBMs (which are nearly all *IDH1/2* WT) have significantly higher *TAGLN2* levels than LGGs (Figure 1). In addition, to our knowledge, no established LGG cell lines currently exist, with the exception of BT142 cells, which do not harbor an *IDH1* wild-type allele [31].

**Figure 4:**
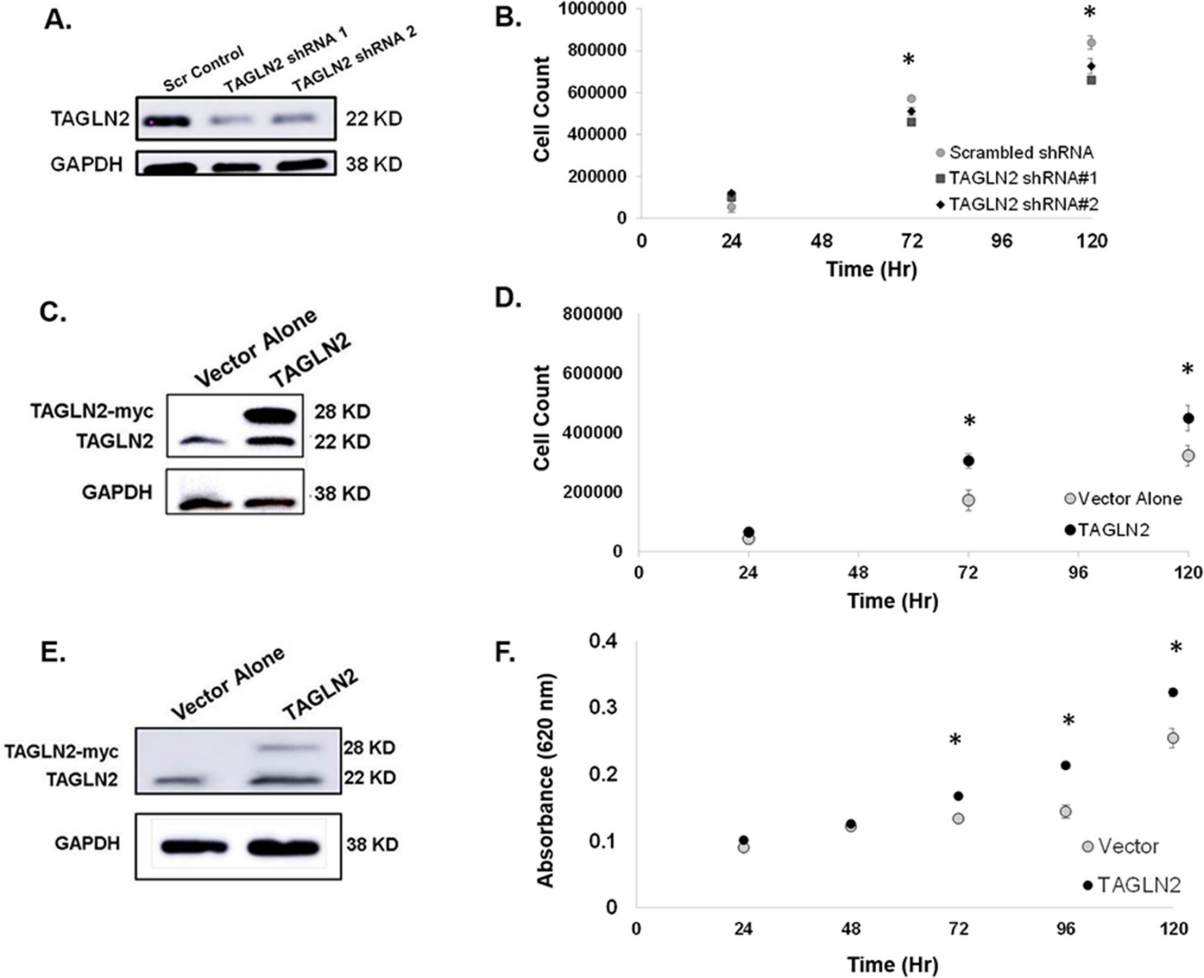
*TAGLN2* regulates proliferation of *IDH1/2* WT GBM cells *in vitro* **(A)** GBM30 neurospheres stably expressing *TAGLN2* shRNA and corresponding scrambled shRNA control were generated and the level of stable *TAGLN2* knock-down detected by Western blot is shown. **(B)** GBM30 neurospheres with stable knock-down of *TAGLN2* or scrambled shRNA control were counted at 24, 72, and 1120 hours after plating. Knock-down of *TAGLN2* resulted in significantly decreased cell counts (p<0.05). **(C)** GBM30 neurospheres and **(D)** U87 MG glioma cells stably overexpressing *TAGLN2* and corresponding vector control were generated and the level of stable *TAGLN2* overexpression was detected by Western blot. Of note, endogenous *TAGLN2* (22 Kda) and exogenous *TAGLN2*-myc (28 kDa) are shown. **(E)** GBM30 neurospheres stably overexpressing *TAGLN2* resulted in significantly increased cell proliferation compared to vector control at 72 and 120 hours (p<0.05). **(F)** U87 MG cells stably overexpressing *TAGLN2* resulted in increased cell proliferation compared to the vector alone. Experiments were performed twice with six replicates each. ^*^, statistically significant difference in proliferation.

GBM30 (primary GBM neurospheres) cells stably expressing *TAGLN2* shRNA or scrambled shRNA control were generated for our knock-down *in vitro* studies. The level of stable knock-down of *TAGLN2* protein was detected by Western blot analysis (Figure 4A). GBM30 neurospheres were trypsinized and counted 24, 72 and 120 hours after plating in the presence of trypan blue for the exclusion of non-viable cells. As shown in Figure 4B, there was a moderate but significant decrease in cell proliferation at 72 and 120 hours in GBM30 cells in which *TAGLN2* expression was markedly knocked down using 2 different *TAGLN2*-shRNA compared to the control shRNA (p<0.05). Similar results were obtained when *TAGLN2* expression was transiently knocked down by RNAi in established GBM cell lines U87 MG and LN18 (Supplemental Figure 2).

Since *TAGLN2*-knockdown compromised the proliferation of GBM cells, we also evaluated whether *TAGLN2* overexpression was sufficient to increase cell proliferation. We generated stable GBM30 and U87 MG cell lines overexpressing *TAGLN2* (tagged with Myc-epitope). Figure 4C and 4E show increases in the level of endogenous (22 kDa) and Myc-tagged exogenous (28 kDa) *TAGLN2* expression in both GBM30 and U87 MG cells, respectively. We counted cells with trypan blue for non-adherent GBM30 neurospheres and performed methylene blue assay for U87 MG cells. In both cell models, *TAGLN2* overexpression resulted in a significant increase in cell proliferation after 72 hours of incubation (p<0.05) (Figure 4D and 4F).

### *TAGLN2* regulates invasion of *IDH1/2* WT GBM cells *in vitro*

**Figure 5:**
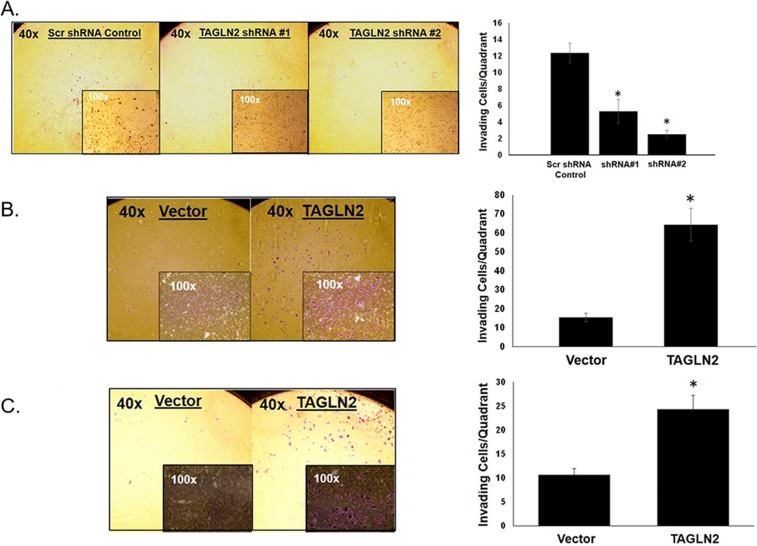
*TAGLN2* regulates invasion of *IDH1/2* WT GBM cells *in vitro* Since *TAGLN2* has been shown to play a role in invasion and metastases of other cancer types, the invasive ability of **(A)** GBM30 neurospheres with stable shRNA-mediated knock-down of *TAGLN2* were compared to their respective scrambled shRNA control. GBM30 cells showed a decrease in average number of cells invading through the matrix after knock-down of *TAGLN2* compared to control. In contrast, **(B)** GBM30 neurospheres and **(C)** U87 MG glioma cells with stable overexpression of *TAGLN2* showed an increase in average number of cells invading through the matrix compared to vector control. Experiments were performed three times with triplicate invasion assays. ^*^, statistically significant difference in invading cells (p<0.05). Photographs are representative images at 40x and 100x magnification.

Since invasion into the surrounding brain parenchyma is a hallmark of gliomas and GBMs that contributes to local recurrence and worse prognosis, we assessed whether *TAGLN2* is involved in invasion by glioma cells. The ability of GBM30 neurospheres to invade through an extracellular matrix after shRNA-mediated stable knock-down of *TAGLN2* was compared to each respective cell type stably transfected with scrambled shRNA control. Figure 5A shows a decreased average number of cells invading through the matrix after knock-down of *TAGLN2* in GBM30 cells, respectively. As previously shown (Figure 4), there were minimal differences in cell proliferation before 24 hours, further suggesting that the number of invading cells were due to invasion capability and not cell proliferation. These experiments were also performed with siRNA-mediated transient knock-down of *TAGLN2* in U87 MG and LN18 cells and similar results were obtained (Supplemental Figure 2).

Since knockdown of *TAGLN2* expression compromised the invasion of GBM cells, we also evaluated whether *TAGLN2* overexpression increased invasion. GBM30 neurospheres and U87 MG cells stably overexpressing *TAGLN2* or vector control were plated on invasion chambers. As shown in Figure 5B and 5C, *TAGLN2* overexpression significantly increased the number of invading cells by approximately 2.5 to three-fold in glioma cells (p<0.05).

### TAGLN2 protein expression is higher in U87 MG (*IDH1/2* WT) parental cells compared to U87 MG cells overexpressing IDH1 R132H mutant protein due to hypomethylation of *TAGLN2* promoter

**Figure 6:**
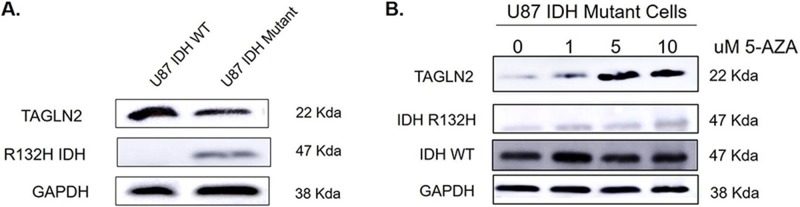
*TAGLN2* protein expression is higher in U87 MG (IDH1/2 WT) parental cells compared to U87 MG cells overexpressing IDH1 R132H mutant protein due to hypomethylation of *TAGLN2* promoter **(A)** *TAGLN2* protein levels were compared in U87 MG *IDH1/2* WT parental cells and a commercially available U87 MG isogenic cell line overexpressing *IDH1* with a heterozygous R132H mutation by Western blot analysis. *TAGLN2* protein was decreased in IDH1 mutant cells compared to *IDH1/2* WT cells. **(B)** U87 MG *IDH1* mutant cells were treated with increasing concentrations of 5-azacytidine (5-AZA) demethylating agent and *TAGLN2* protein was evaluated by Western blot. 5-AZA resulted in increasing *TAGLN2* protein levels expression in a dose-dependent manner.

Since *TAGLN2* was found to be expressed at significantly lower mRNA and protein levels in *IDH1/2* mutant gliomas from both institutional and TCGA patient cohorts, we confirmed these findings *in vitro* by evaluating *TAGLN2* protein levels in a commercially available U87 MG isogenic cell line overexpressing *IDH1*R132H mutant protein (ATCC, Manassas, VA), which will be referred to as *IDH1* mutant U87 MG cells. We compared levels of *TAGLN2* protein in U87 MG cells overexpressing mutant *IDH1* to U87 *IDH1/2* WT MG parental cells by Western blot. Similar to our patient-derived glioma tissues, cell lines expressing mutant *IDH1* had decreased *TAGLN2* protein levels compared to U87 MG *IDH1/2* WT glioma cells (Figure 6A). Since our methylation data in glioma patients suggested that *TAGLN2* expression might be silenced in *IDH1/2* mutant LGGs by promoter hypermethylation, we confirmed these results *in vitro* by treating *IDH1* mutant U87 MG cells with 5-aza-2′-deoxycytidine (5-AZA), an inhibitor of DNA methyltransferase that results in DNA demethylation [32]. Cells were treated with increasing concentrations of 5-AZA (1, 5 and 10 μM) for 96 hours and *TAGLN2* protein levels were evaluated by Western blot analysis. As shown in Figure 6B, treatment with 5-AZA resulted in increased *TAGLN2* protein expression in an almost dose-dependent manner in *IDH1* mutant U87 MG cells. Treatment of *IDH1/2* WT U87 MG with 5-AZA did not change *TAGLN2* protein levels (data not shown). These results suggest that *TAGLN2* expression may be decreased due to its promoter hypermethylation-mediated transcriptional downregulation in *IDH1/2* mutant gliomas.

## DISCUSSION

It has been well-established that *IDH1/2* WT gliomas demonstrate worse prognoses compared to gliomas with *IDH1/2* mutations [4]. While multiple explanations have been proposed, the underlying biological mechanisms responsible for these survival differences remain poorly understood. In this study, we performed mRNA and proteomic profiling of *IDH1/2* WT versus *IDH1/2* mutant Grade II and III gliomas in order to better understand gene expression differences contributing to worse overall survival in *IDH1/2* WT gliomas. Our analyses identified *TAGLN2* as one of the significantly up-regulated genes in *IDH1/2* WT compared to *IDH1/2* mutant low(er) grade gliomas. Subsequently, we tested the hypothesis that increased *TAGLN2* expression may contribute to worse prognoses in *IDH1/2* WT gliomas by investigating the role and regulation of *TAGLN2* regulation in both LGGs and GBMs.

While in agreement with a recent study showing that *TAGLN2* is a poor prognostic marker for gliomas, our study is the first to report that *TAGLN2* is a negative prognostic factor associated with *IDH1/2* WT gliomas and its regulation in gliomas is highly dependent on *IDH1/2* mutation status. Previous studies have demonstrated that reorganization of the methylome and the resulting hypermethylator phenotype in gliomas is established by the presence of mutant *IDH1/2* [33, 34]. However, the individual genes involved in G-CIMP and their associated effects on oncogenesis remain uncertain. We are the first to identify *TAGLN2* as a gene likely to be involved in the hypermethylator phenotype and further show that *TAGLN2* undergoes epigenetic regulation in *IDH1/2* mutant gliomas. Our global methylation analysis of low(er) grade gliomas from our institutional cohort showed that CpG sites corresponding to the *TAGLN2* promoter are more highly methylated in *IDH1/2* mutant compared to *IDH1/2* WT gliomas. Therefore, our data suggest that epigenetic silencing by promoter hypermethylation likely accounts for decreased *TAGLN2* mRNA and protein levels observed in *IDH1/2* mutant gliomas. Our *in vitro* studies confirmed that overexpression the protein of *IDH1*R132H in U87 MG GBM cells resulted in decreased *TAGLN2* expression compared to U87 MG *IDH1/2* WT parental cells and further showed that treatment of *IDH1* mutant cells with 5-AZA, a DNA methyltransferase inhibitor, rescued *TAGLN2* expression after demethylation. Such an association between the *IDH1/2* mutation and *TAGLN2* expression is also demonstrated by our multivariate analysis that showed *TAGLN2* as a negative prognostic factor, though highly dependent on *IDH1/2* mutation status.

While our study is limited by the availability of only one established heterozygous *IDH1* mutant glioma cell line, the epigenetic regulation of *transgelin*, a homologue of *TAGLN2*, has been reported in multiple other cancer types. While these homologues share 64% amino acid sequence homology and similar functions, *transgelin* is mainly expressed in smooth muscle cells and fibroblasts whereas *TAGLN2* is expressed primarily in epithelial cells. *Transgelin* has been reported to be regulated by promoter hypermethylation in breast [35], colorectal [36], malignant peripheral nerve sheath tumors [37] and hepatocellular carcinomas [38]. Moreover, promoter methylation has also been shown to regulate *transgelin* expression during normal physiologic processes, including epigenetic silencing of *transgelin* in endometrial stroma during reorganization of the actin cytoskeleton in preparation for pregnancy [39]. Similar to our data, Logan et al. [39] showed that treatment with 5-AZA demethylating agent rescued *transgelin* expression in endometrial stromal cells.

While *TAGLN2* expression appears to be regulated at the epigenetic level in *IDH* mutant gliomas, we also found significant differences in *TAGLN2* mRNA expression among different grades of *IDH* WT gliomas, suggesting that *TAGLN2* expression may also be regulated during glioma progression in a 2HG-independent fashion. Our results are in agreement with a recent study that found higher levels of multiple *transgelin* peptides in stem cells isolated from high grade gliomas compared to low grade gliomas [40]. Moreover, Han et al. [22] reported that *TGF-β* signaling increases *TAGLN2* expression, which in turn regulates the epithelial-mesenchymal transition (EMT) leading to acquisition of a mesenchymal phenotype and increased cell invasion [41]. Taken together, these results suggest that *TAGLN2* may be involved in glioma progression.

A more invasive phenotype is believed to contribute to worse prognoses in *IDH1/2* WT LGGs compared to *IDH1/2* mutant gliomas [42–46], among other proposed mechanisms. Infiltration into the surrounding brain parenchyma is a hallmark of gliomas and GBMs that allows cancer cells to escape surgical resection and radiation, often resulting in local recurrence after aggressive therapy. Beiko et al. [42] concluded that *IDH* mutant astrocytomas were more amenable to gross surgical resection due to minimal invasion compared to *IDH* WT astrocytomas [42], therefore reducing the risk of local recurrence. Moreover, Price et al. [44] found that *IDH* mutant GBMs were less invasive than *IDH* WT GBMs based on MRI diffusor-tensor imaging, a technique believed to delineate tumor margins more accurately than conventional MRI. Another study further showed *IDH* mutant cells to have a less invasive phenotype than *IDH* WT cells *in vivo* and *in vitro* [45]. Our results show that *TAGLN2* significantly contributes to invasion and proliferation in *IDH* WT gliomas, however the mechanism remains poorly understood.

Glioma cell invasion is a multi-step process that requires several changes to occur within the cell, including gaining cell motility through reorganization of the cytoskeleton, detachment from the primary tumor, adhesion to the extracellular matrix (ECM) and degradation of the ECM [47, 48]. However, the role of *TAGLN2* in this complex process remains to be elucidated. A recently published study provided evidence that *TAGLN2* is capable of regulating the actin cytoskeleton in response to extracellular signals when localized to the cell membrane in airway smooth muscle cells [49]. Indeed, *TAGLN2* is believed to contribute to invasion by remodeling the actin cytoskeleton and forming cell protrusions, referred to as invadopodia, that promote cell motility [30]. Other studies have demonstrated that *TAGLN2* and its homologues have the ability to localize to the nucleus and regulate expression of invasion-related genes that facilitate detachment from tumor cells as well as adhesion to and degradation of the ECM [22]. *Transgelin* and its homologues have been shown to regulate expression of critical invasion-related genes, including adherens junctions (*E-cadherin* and *B-catenin*), tight junctions (*occludin*), intermediate filament proteins (*vimentin*), adhesion glycoproteins (*fibronectin*), proteases (*MMP-9, MMP-2*) and chemokine receptors (*CXCR*4) [17, 21, 30, 50–52]. Furthermore, Han et al. [30] suggested that *TAGLN2* may promote EMT and invasion in glioma cells by up-regulating expression of mesenchymal markers, such as *N-cadherin, Snail, Slug and Twist*. *TAGLN2* therefore appears to regulate glioma invasion at multiple levels.

In summary, we have shown that *TAGLN2* expression is silenced by promoter hypermethylation and therefore likely involved in the glioma hypermethylator phenotype of *IDH1/2* mutant gliomas. Moreover, we have shown that *TAGLN2* is highly expressed in *IDH1/2* WT gliomas and GBMs and appears to function as an oncogene. This differential regulation of *TAGLN2* provides further insight into the genetic, epigenetic and oncogenic differences between *IDH1/2* WT and mutant gliomas. Taken together, our data demonstrate that *TAGLN2* protein functions as an oncogene and may serve as a novel therapeutic target for effectively treating *IDH1/2* WT LGG and GBM.

## MATERIALS AND METHODS

### Glioma databases

Sixty-seven Grade II and III formalin-fixed paraffin embedded (FFPE) glioma specimens in our institutional cohort were provided by the University of Freiburg. Corresponding clinical data including age at diagnosis, gender, KPS, histology and overall survival (OS) were also provided. OS was defined as the time from pathologic glioma diagnosis (date of surgery) to date of death or last documented date of follow-up. Clinical data for Grade II, III and IV gliomas included in The Cancer Genome Atlas (TCGA) research database as well as their corresponding mRNA expression, promoter methylation, *IDH1/2* mutation and 1p/19q co-deletion analysis were obtained from the publicly available data portal at https://cancergenome.nih.gov.

Briefly, *IDH1/2* mutation status for our institutional cohort was determined by next-generation sequencing with a customized IonTorrent panel (ThermoFisher Scientific, Waltham, MA) that included amplicons covering the coding regions (>98% of bases) of *IDH1* and *IDH2* [53, 54]. Patients were deemed *IDH1/2* mutant if mutations were present at *IDH1*R132 or *IDH2*R172 positions. *1p/19q* co-deletion status was determined by the Infinium Human Methylation 450K array (Illumina) and/or the Affymetrix Oncoscan v2.0 platform (Thermofisher Scientific, Waltham, MA). A reference from the CopyNumber450kData package was used. For *1p/19q* status, the ranges chr1 15865-100000000 and chr19 40000000-59095508 bp, respectively were analyzed. A median log R ratio of −0.2 was used to call deletions of each of these regions. Samples were omitted if deemed of poor quality (due to large variation in the by probe log R ratios) based on the plots. Co-deletion status was determined by the Nexus calls for the Oncoscan data, as well as visual inspection of the segmentation plots for each sample.

### Microarray analysis

Total RNA was extracted from FFPE glioma tissues with the miRNAeasy kit (Qiagen, Hilden, Germany). Only RNA with a 260/280 ratio of at least 1.8 measured by Nanodrop was used for the microarray analysis. cRNA was hybridized to the Affymetrix HTA ClariomD array (Thermofisher Scientific, Waltham, MA). Normalizaton (SST-RMA) and summarization were carried out using the Affymetrix transcriptome analysis console software. Differential gene expression between *IDH1/2* WT and *IDH1/2* mutant gliomas was determined by LIMMA analysis [55]. The cox regression model was employed to identify associations between gene expression and overall survival.

### Proteomics

Proteins were comparatively evaluated between *IDH1/2* mutant and WT tumors by mass spectrometry as previously described in Bassett et al. [56]. Briefly, proteins were digested with trypsin and LysC and resulting peptides were analyzed by mass spectrometry (completed by CWRU Center for Proteomics). LC-MS/MS data collection was performed using a UPLC system (NanoAcquity, Waters) interfaced to an Orbitrap ProVelos Elite MS system (ThermoFisher Scientific). All peptide precursor ions across all chromatographic analyses were clustered using Rosetta Elucidator software. Peptide and protein identifications were integrated from the protein database search engine output (MASCOT, Matrix Science Inc.) [56].

### Methylation

DNA was extracted from FFPE tissues using a combination of Recoverall Total Nucleic Acids Isolation (ThermoFisher Scientific) and Epicentre Masterpure DNA Purification (Illumina) kits. DNA quantity was assessed using the Qubit dsDNA High Sensitivity Assay Kit (ThermoFisher Scientific). Approximately 250 ng of DNA was submitted to the University of Southern California Epigenome Center (Los Angeles, CA) and run on the Illumina Human Methylation-450K Array. Data were processed using the R package “minfi” with hg19 annotation. Data were Noob normalized and M-value [57] transformed. Probes failing in > 10% (by Illumina’s detection p-value) of samples were removed (n=1301). Samples failing across > 10% of probes were removed (n=0). SNP containing probes were removed (n=17, 534).

### Cell culture

U87 MG high grade glioma cell lines (American Type Culture Collection (ATCC), Manassas, VA, USA) were maintained in DMEM media (Gibco, Life Technologies, Gaithersburg, MD) with 10% Fetal Bovine Serum (FBS) and 1% penicillin/streptomycin. An isogenic line derived from overexpression of *IDH1R132H* in U87 MG parental cells was purchased from ATCC (Manassas, VA, USA). This cell line has a heterozygous C395G>A knock-in mutation encoding *IDH1R132H* protein expression generated by using the CRISPR/Cas9 gene editing technology. GBM30 glioma neurospheres were a generous gift from Balveen Kaur’s lab and have been previously characterized [58, 59]. They were cultured in neurobasal medium (Gibco, Life Technologies, Gaithersburg, MD) supplemented with 20% B27 (Invitrogen, Carlsbad, CA), 100 ug/ml penicillin/streptomycin, 20 ng/ml epidermal growth factor (EGF), and 20 ng/ml basic fibroblast growth factor. Cells were maintained at 37 °C in 5% CO_2_.

### Development of stable cell lines

*Stable TAGLN2 Knock-down:* Short hairpin RNA (shRNA) targeting *TAGLN2* were transfected into cells using Lipofectamine 2000 (ThermoFisher Scientific, Waltham, MA) per the manufacturer’s protocol and *TAGLN2* knock-down efficiency was measured 48 hours after transfection. For GBM30 cells, shRNA targeting *TAGLN2* was purchased from Origene with the following sequences: shRNA#1: CTGTGTGCAGCGGACGCTGATGAATCTGG and shRNA#2: GGCGTCTCAGGCAGGCATGACTGGCT ACG. Control cells were transfected with scrambled shRNA control in the pGFP-V-RS shRNA vector (Origene, Rockville, MD). shRNA #3 targeting human *TAGLN2* was purchased from Sigma and transfected into U87 MG cells with the following sequence: CCGGGAACGTGATCGGGTTACAGATCTCGAGATCTGTAACCCGATCACGTTCTTTTTTG (Sigma, St. Louis, MO). Transfection with MISSION pLKO.1-puro Non-Mammalian shRNA Control Plasmid DNA was used for the corresponding scrambled shRNA control (Sigma, St. Louis, MO). Different *TAGLN2*-targeted shRNAs with greater knock-down efficiency were required for GBM30 cells due to their lower transfection rates. Stable cell lines were maintained in selection media supplemented with puromycin (2 μg/mL) (Sigma, St. Louis, MO).

### Stable *TAGLN2* overexpression

Human *TAGLN2* (Myc-DDK-tagged) in pCMV6-entry vector (Origene, Rockville, MD) was transfected into GBM30, U87 MG *IDH1/2* WT and U87 MG glioma cells overexpressing mutant *IDH1R132H* using Lipofectamine 2000 (Thermo Scientific, Waltham, MA) according to the manufacturer’s protocol. Control cells were transfected with pCMV6-entry vector alone. Stable cell lines were maintained in selection media supplemented with G418 at 400μ/mL (Sigma, St. Louis, MO).

### Western blot

Cells (5 × 10^5^) were seeded in 60 mm plates, washed with PBS, trypsinized and transferred to a microcentrifuge tube. After centrifuging and washing in PBS, cells were lysed in RIPA buffer (Sigma, St. Louis, MO) containing phosphatase and protease inhibitor cocktail and 1 mmol/L phenylmethanesulfonyl fluoride (Thermo Scientific, Waltham, MA). Protein concentrations were measured using the BSA protein assay (Pierce, Thermo Scientific, Waltham, MA) per protocol. Equal amounts of protein were separated with 10% SDS-PAGE gel electrophoresis and transferred to Hybond ECL nitrocellulose membranes (GE Healthcare, Chicago, IL). After blocking for 1 hour in Tris-buffered saline containing 0.1% Tween 20 and 5% milk, the membranes were probed with various primary antibodies: anti-*TAGLN2* polyclonal antibody (Abcam, Cambridge, United Kingdom), anti-*IDH1 R132H* mutant antibody (Cell Signaling Technologies, Danvers, MA) and anti*-glyceraldehyde-3-phosphate dehydrogenase (GAPDH)* monoclonal antibody (American Research Products, Waltham, MA). Membrane was then probed with secondary anti-rabbit IgG conjugated to horseradish peroxidase (HRP) and secondary anti-mouse IgG-HRP (Cell Signaling Technologies, Danvers, MA). Immunoreactivity was detected by enhanced chemiluminescent (ECL) kit (Amersham Biosciences Co., Little Chalfont, United Kingdom).

### Proliferation assays

U87 MG stable cells (2×10^3^ cells) were seeded in a 96 well-plate. Six replicates were seeded for each of five time points, including 24, 48, 72, 96 and 120 hours. At each time point, cells were washed with PBS twice and fixed with 4% paraformaldehyde for 20 minutes. Cells were stained with 1% methylene blue for 25 minutes. Methylene blue was dissolved in an acetic acid solution and absorbance at 620 nm was measured using a spectrophotometer. Since the neurospheres are non-adherent, we performed cell proliferation assays by counting cells at 24, 72 and 120 hours using Trypan blue to exclude non-viable cells. Briefly, 1×10^5^ dissociated GBM30 cells were seeded in a 24 well plate with serum-free media for three different time points, including 24, 72 and 120 hours. At each time point, cells were dissociated, counted and compared between the vector control and the stable cell lines. Cell count (absorbance) at each time point was compared between stable cell lines with *TAGLN2* knock-down/overexpression relative to their respective control cells using t-test (p<0.05). Experiments were performed three times with 4 replicates.

### Invasion assay

Matrigel invasion chambers (Corning, Corning, NY) were incubated in serum-free media two hours before seeding cells. 2 × 10^5^ U87 MG and GBM30 cells were seeded onto matrigel invasion chambers. Cells were incubated in chamber over 24 hours at 37 °C in 5% CO_2_. After incubation, cells were fixed and stained in 0.5% crystal violet with methanol for 20 minutes and washed in PBS. Average number of invading cells in each quadrant was averaged and compared between stable cell lines with *TAGLN2* knock-down/overexpression relative to their respective control cells using t-test (p<0.05). Experiments were performed three times with duplicates. Photos of stained cells in invasion matrix were obtained using the EVOS XL Core microscope (ThermoFisher Scientific, Waltham, MA).

### Statistics

For two group univariable survival comparisons, Kaplan Meier curves were plotted and the log-rank test applied. For multivariable and survival associations with continuous variables, Cox regression was used. For two group comparisons, two sample t-tests were employed, and for multiple group analyses ANOVA was used followed by pairwise comparisons using Tukey’s post-hoc test. For high dimensional analyses, FDR < 0.1 was considered significant, while for conformational analyses a raw P<0.05 was considered to be statistically significant. Heat maps were produced using Ward clustering on correlations.

## SUPPLEMENTARY MATERIALS FIGURES



## Abbreviations

(LGG): Low(er) grade gliomas
(GBM): glioblastoma
(TAGLN2): transgelin-2
(IDH): isocitrate dehydrogenase
(WT): wild-type
(WHO): World Health Organization
(α-KG): α-ketoglutarate
(2-HG): 2-hydroxyglutarate
(TAGLN1): transgelin-1
(TAGLN3): transgelin-3
(5-AZA): 5-azacytidine
