# Supplementary material for: Oncogenic transgelin-2 is differentially regulated in isocitrate dehydrogenase wild-type vs. mutant gliomas
Source: Oncotarget. 2018 Dec 14;9(98):37097–111. doi: 10.18632/oncotarget.26365 (PMC6324682; doi:10.18632/oncotarget.26365)
Supplement: Supplementary file 1 [file oncotarget-09-37097-s001.pdf]

Oncogenic *transgelin-2* is differentially regulated in *isocitrate dehydrogenase* wild-type vs. mutant gliomas

SUPPLEMENTARY MATERIALS

Peptide Sequence	Protein	Fold Change IDH WT/Mutant	P-Value	FDR
NFSDNQLQEGK	TAGLN2	2.32	1.49×10^{-3}	0.49
GASQAGMTGYGMPR	TAGLN2	1.93	9.01×10^{-3}	0.67
TLMNLGGLAVAR	TAGLN2	1.94	1.65×10^{-2}	0.75

Supplementary Figure 1: Proteomics analysis of an institutional validation LGG cohort (7 IDH1/2 WT vs. 23 IDH1/2 mutant) identified 36 differentially regulated proteins in common with our discovery cohort ($p<0.05$). TAGLN2 was among the 36 differentially regulated proteins. Three peptides corresponding to TAGLN2 were significantly up-regulated in *IDH1/2* WT compared to *IDH1/2* mutant gliomas.

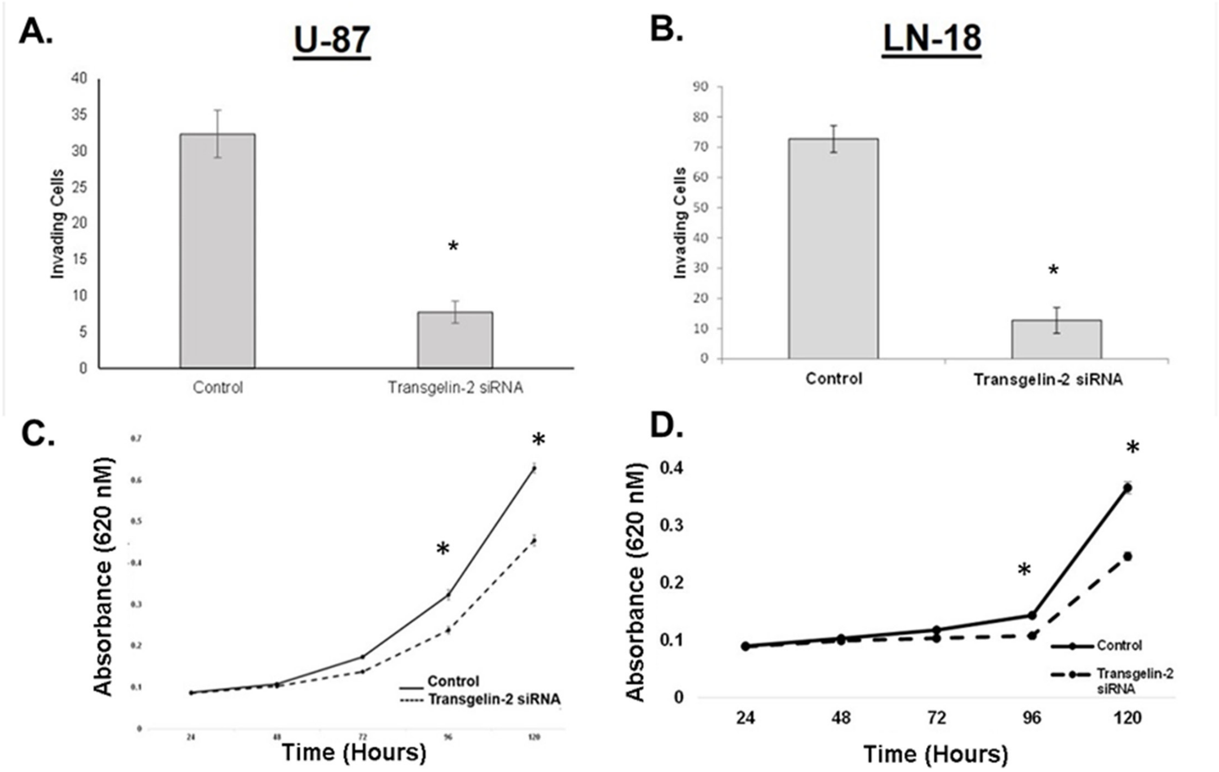

Supplementary Figure 2: Silencing of TAGLN2 in IDH1/2 WT glioma cells resulted in decreased invasion and proliferation. *TAGLN2* levels were transiently knocked-down with *TAGLN2* siRNA in U87 MG and LN18 glioma cells. **(A and B)** Invasion was significantly decreased 24 hours after TAGLN2 knock-down ($p<0.05$). **(C and D)** Cell proliferation detected by methylene blue assay was significantly decreased 96 and 120 hours after TAGLN2 knock-down ($p<0.05$).
